# Supplementary material for: Comprehensive lipidomic analysis reveals regulation of glyceride metabolism in rat visceral adipose tissue by high-altitude chronic hypoxia
Source: PLoS One. 2022 May 6;17(5):e0267513. doi: 10.1371/journal.pone.0267513 (PMC9075645; doi:10.1371/journal.pone.0267513)
Supplement: S1 File — (DOC) [file pone.0267513.s001.doc]

**Supporting information**

**S1 Fig1.** Typical base peak intensity chromatograms for the visceral adipose tissue of rats, derived from QC samples in positive ion mode.


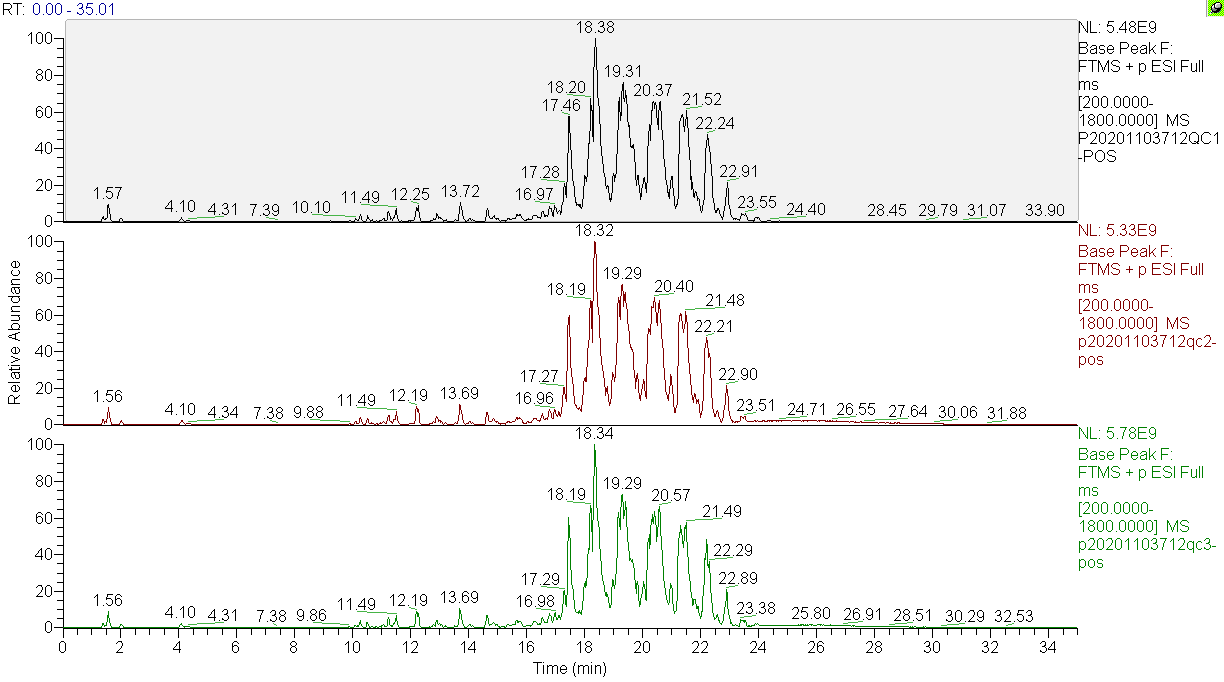


**S1 Fig****2.** Typical base peak intensity chromatograms for the visceral adipose tissue of rats, derived from QC samples in negative ion mode.


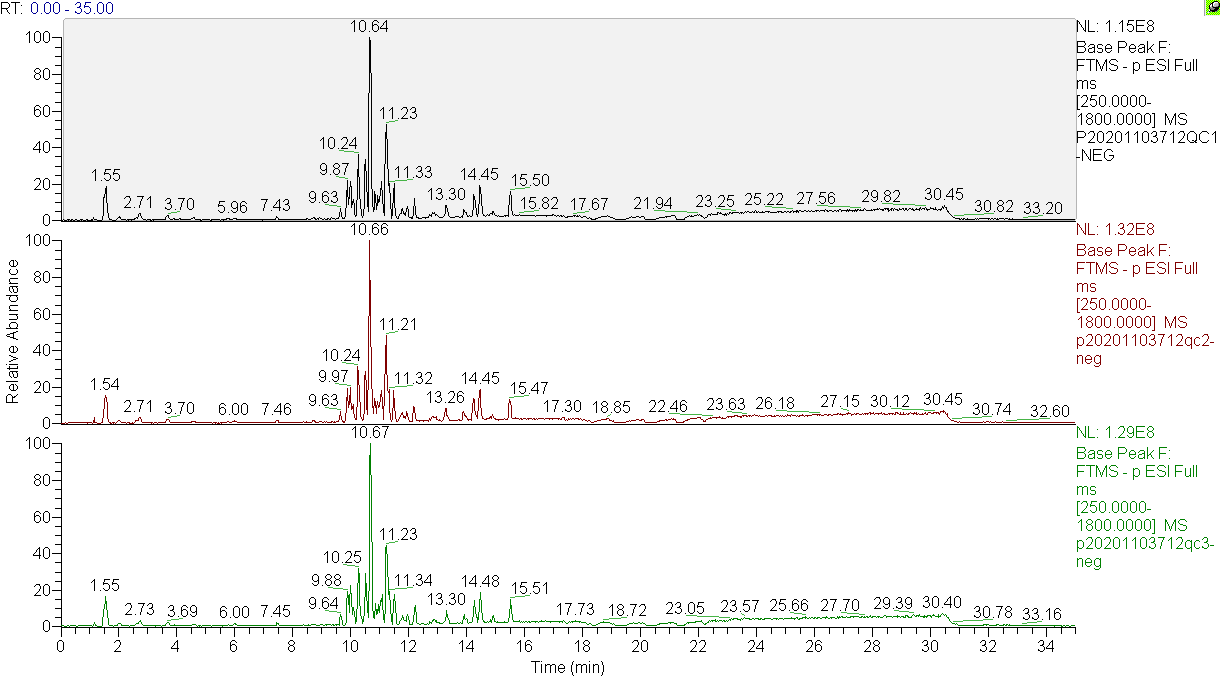


**S1 Fig3.** Correlation map of QC samples


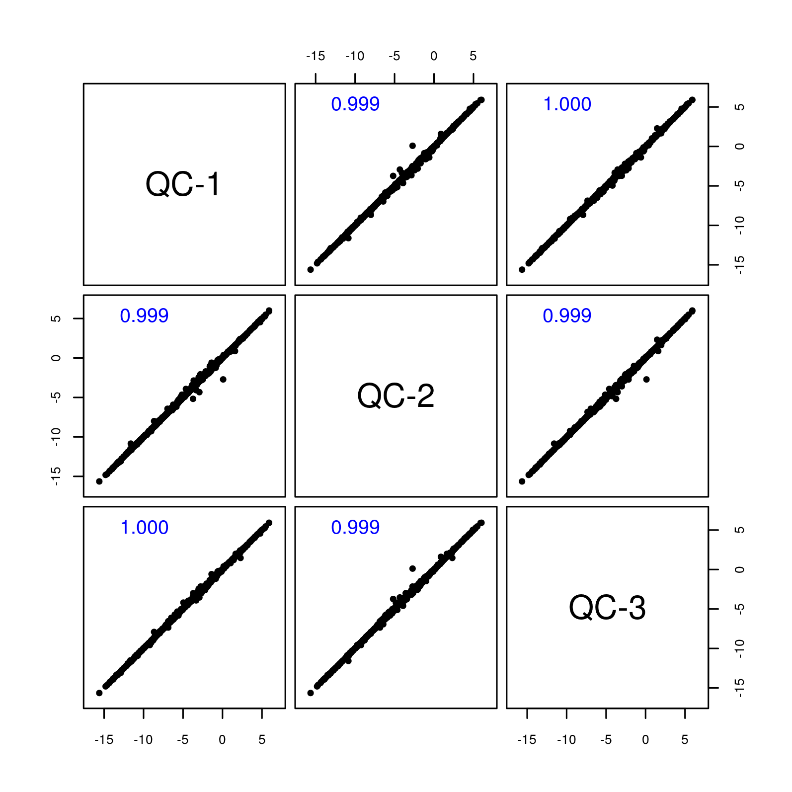


**S1 Fig4.** PCA analysis of all samples


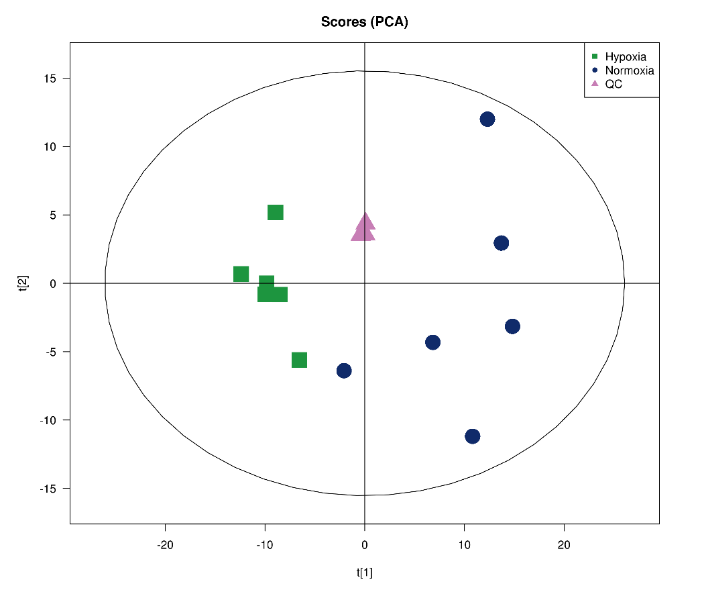


**S1 Fig5.** PLS-DA score plot


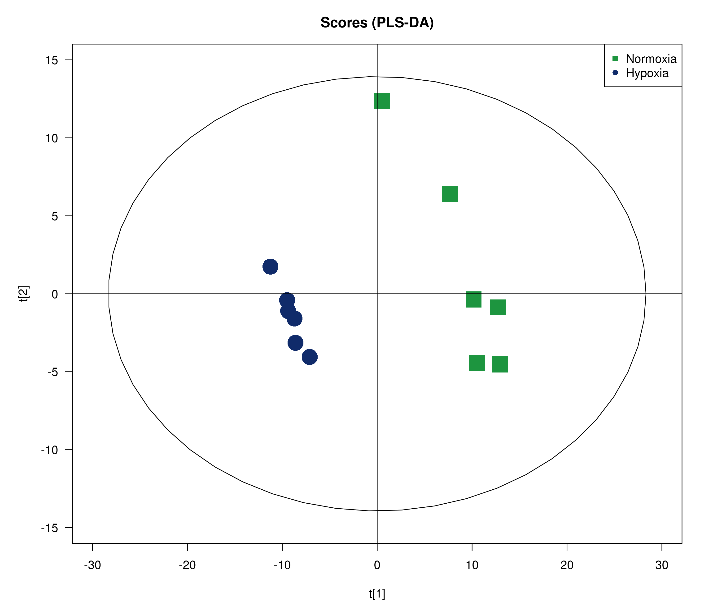


**S1 Fig6.**  Hotelling's T2 test of all samples.


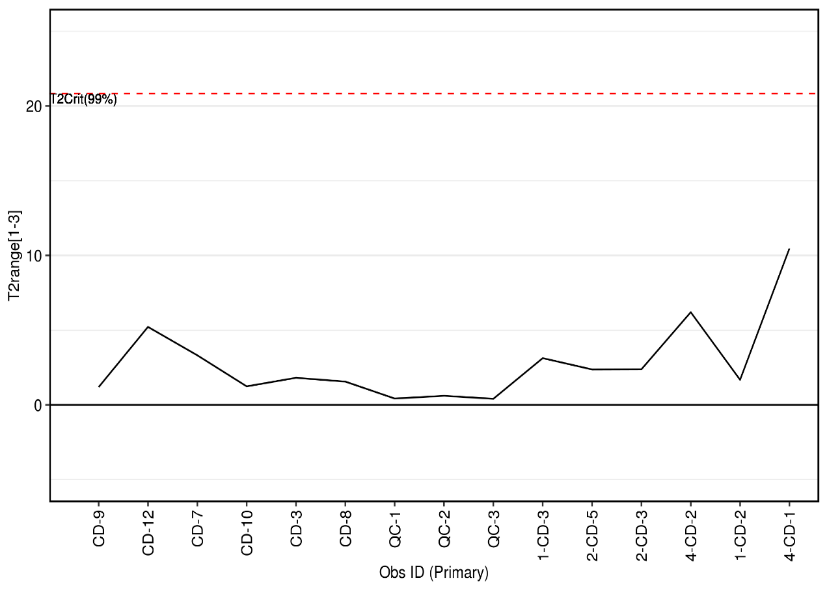


**S1 Fig7.**  MCC diagram of QC samples.


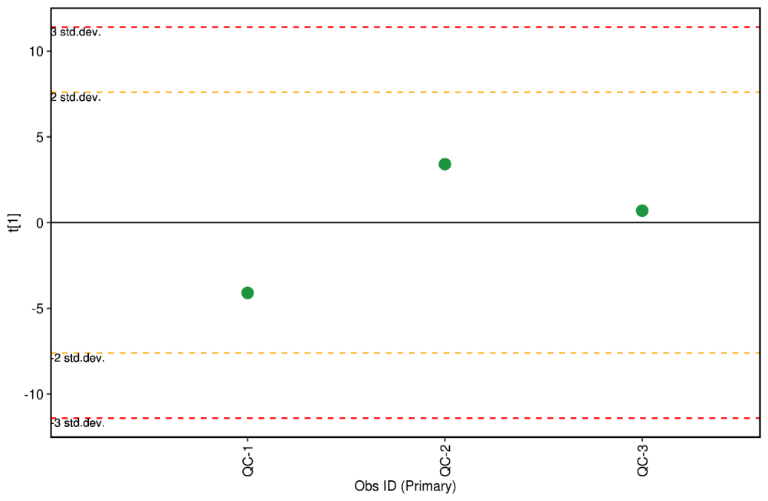


**S1 Fig8.**  Relative standard deviation of QC samples.


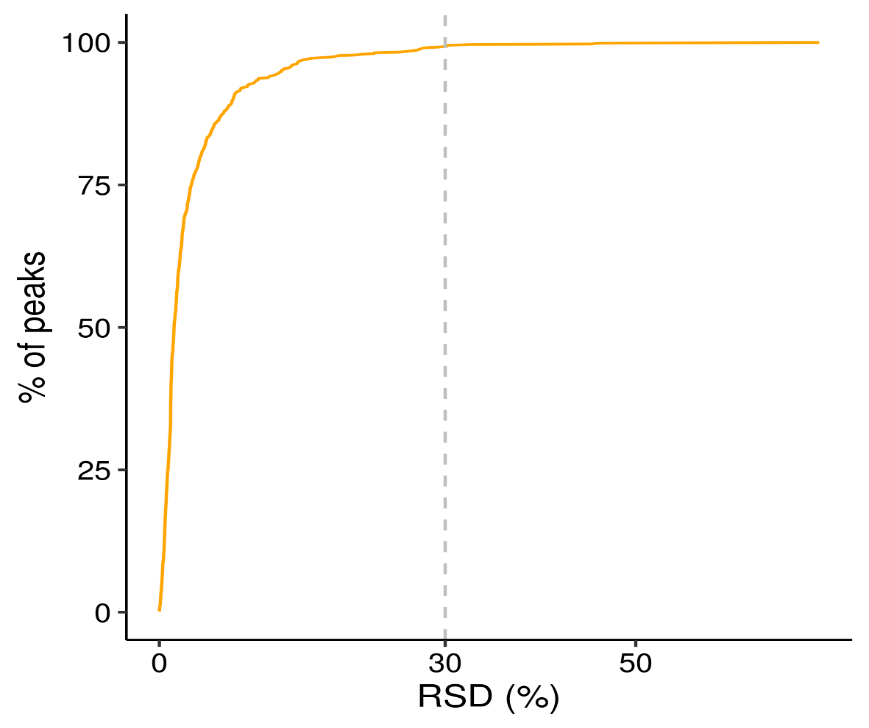


**S1 Table1**  PCA Model Parameters between normoxia group and hypoxia group.

| Sample Group | A | R^2^X（cum） |
| --- | --- | --- |
| QC | 3 | 0.576 |
| Normoxia VS Hypoxia | 3 | 0.6 |

**S1 Table** 2 PLS—DA Model evaluation Parameters between normoxia group and hypoxia group.

| Sample Group | A | R^2^X（cum） | R^2^Y（cum） | Q^2^（cum） |
| --- | --- | --- | --- | --- |
| Normoxia VS Hypoxia | 3 | 0.521 | 0.998 | 0.879 |

**S1 Table 3** OPLS—DA Model evaluation Parameters between normoxia group and hypoxia group.

| Sample Group | A | R^2^X（cum） | R^2^Y（cum） | Q^2^（cum） |
| --- | --- | --- | --- | --- |
| Normoxia VS Hypoxia | 1+2 | 0.521 | 0.998 | 0.882 |

**S1 Table 4** Differences in lipids (TGs and DGs) between normoxia group and hypoxia group.

| **name** | **Lipid Ion** | **Ion Formula** | **CalMz** | **RT-(min)** | **VIP** | **Fold Change** | **P-value** |
| --- | --- | --- | --- | --- | --- | --- | --- |
| 113POS | TG(6:0/14:0/16:1)+NH4 | C39 H76 O6 N1 | 654.5667 | 13.5286 | 1.2238 | 12.2091 | 0.0159 |
| 115POS | TG(4:0/16:0/16:0)+NH4 | C39 H78 O6 N1 | 656.5824 | 14.5883 | 1.1619 | 9.3443 | 0.0099 |
| 139POS | TG(10:0/10:0/18:2)+NH4 | C41 H78 O6 N1 | 680.5824 | 13.6980 | 1.9416 | 5.6982 | 0.0196 |
| 141POS | TG(6:0/16:0/16:1)+NH4 | C41 H80 O6 N1 | 682.5980 | 14.6126 | 2.2609 | 8.4332 | 0.0155 |
| 168POS | TG(6:0/16:0/18:2)+NH4 | C43 H82 O6 N1 | 708.6137 | 14.6358 | 1.8864 | 7.2810 | 0.0149 |
| 170POS | TG(6:0/16:0/18:1)+NH4 | C43 H84 O6 N1 | 710.6293 | 15.6010 | 1.5242 | 13.2948 | 0.0231 |
| 171POS | TG(16:0/8:0/16:0)+NH4 | C43 H86 O6 N1 | 712.6450 | 16.6512 | 1.0514 | 13.4495 | 0.0218 |
| 207POS | TG(6:0/18:1/18:2)+NH4 | C45 H84 O6 N1 | 734.6293 | 14.6603 | 1.1942 | 7.7381 | 0.01961 |
| 209POS | TG(16:0/8:0/18:2)+NH4 | C45 H86 O6 N1 | 736.6450 | 15.6422 | 2.0101 | 9.3209 | 0.0407 |
| 211POS | TG(16:0/8:0/18:1)+NH4 | C45 H88 O6 N1 | 738.6606 | 16.6762 | 2.0272 | 13.5499 | 0.0270 |
| 215POS | TG(16:0/12:0/14:0)+NH4 | C45 H90 O6 N1 | 740.6763 | 17.8065 | 1.3456 | 11.5866 | 0.0195 |
| 252POS | TG(8:0/18:1/18:2)+NH4 | C47 H88 O6 N1 | 762.6606 | 15.6781 | 1.6020 | 8.6592 | 0.0310 |
| 257POS | TG(16:0/10:1/18:1)+NH4 | C47 H90 O6 N1 | 764.6763 | 17.1455 | 1.1883 | 7.8039 | 0.0201 |
| 258POS | TG(16:0/10:0/18:2)+NH4 | C47 H90 O6 N1 | 764.6763 | 16.7744 | 2.6865 | 8.4602 | 0.0380 |
| 264POS | TG(16:0/10:0/18:1)+NH4 | C47 H92 O6 N1 | 766.6919 | 17.8290 | 2.7937 | 11.2469 | 0.0246 |
| 271POS | TG(16:0/14:0/14:0)+NH4 | C47 H94 O6 N1 | 768.7076 | 19.0008 | 1.1537 | 7.9421 | 0.0362 |
| 314POS | TG(10:0/18:1/18:2)+NH4 | C49 H92 O6 N1 | 790.6919 | 16.8106 | 2.3499 | 7.2999 | 0.0330 |
| 319POS | TG(16:0/12:0/18:2)+NH4 | C49 H94 O6 N1 | 792.7076 | 17.9458 | 3.9355 | 7.9659 | 0.0174 |
| 322POS | TG(16:0/14:0/16:1)+NH4 | C49 H96 O6 N1 | 794.7232 | 19.0216 | 3.3927 | 9.0432 | 0.0266 |
| 343POS | TG(15:0/14:0/18:2)+NH4 | C50 H96 O6 N1 | 806.7232 | 18.5642 | 1.2751 | 8.3799 | 0.0266 |
| 347POS | TG(15:0/16:0/16:1)+NH4 | C50 H98 O6 N1 | 808.7388 | 19.6304 | 1.4751 | 9.2691 | 0.0159 |
| 364POS | TG(16:1/14:1/18:2)+NH4 | C51 H94 O6 N1 | 816.7075 | 16.9629 | 1.8399 | 5.2673 | 0.0179 |
| 366POS | TG(16:1/16:1/16:1)+NH4 | C51 H96 O6 N1 | 818.7232 | 17.9962 | 4.6115 | 6.7509 | 0.0246 |
| 367POS | TG(16:0/14:0/18:3)+NH4 | C51 H96 O6 N1 | 818.7232 | 18.2532 | 1.7384 | 6.4996 | 0.0250 |
| 368POS | TG(16:0/14:0/18:2)+NH4 | C51 H98 O6 N1 | 820.7388 | 19.0988 | 4.8918 | 6.1387 | 0.0408 |
| 371POS | TG(16:0/14:0/18:1)+NH4 | C51 H100 O6 N1 | 822.7545 | 20.1869 | 3.5502 | 5.5754 | 0.0405 |
| 389POS | TG(18:2/13:0/18:2)+NH4 | C52 H96 O6 N1 | 830.7232 | 17.5958 | 1.4689 | 8.2563 | 0.0022 |
| 395POS | TG(15:0/16:0/18:3)+NH4 | C52 H98 O6 N1 | 832.7388 | 18.6286 | 2.3132 | 6.6816 | 0.0206 |
| 399POS | TG(15:0/16:0/18:2)+NH4 | C52 H100 O6 N1 | 834.7545 | 19.7764 | 1.0273 | 4.0702 | 0.0452 |
| 405POS | TG(15:0/16:0/18:1)+NH4 | C52 H102 O6 N1 | 836.7701 | 20.8111 | 1.4315 | 3.0665 | 0.0167 |
| 421POS | TG(14:0/18:2/18:2)+NH4 | C53 H98 O6 N1 | 844.7388 | 18.7514 | 2.2339 | 7.7852 | 0.0119 |
| 427POS | TG(16:0/16:1/18:2)+NH4 | C53 H100 O6 N1 | 846.7545 | 19.1662 | 5.1974 | 4.9670 | 0.0370 |
| 428POS | TG(16:0/16:0/18:3)+NH4 | C53 H100 O6 N1 | 846.7545 | 19.4741 | 1.0542 | 4.6992 | 0.0424 |
| 429POS | TG(16:0/16:1/18:2)+NH4 | C53 H100 O6 N1 | 846.7545 | 20.0118 | 1.4272 | 8.8431 | 0.0322 |
| 433POS | TG(16:0/16:0/18:2)+NH4 | C53 H102 O6 N1 | 848.7701 | 21.6396 | 1.4545 | 8.6155 | 0.0174 |
| 438POS | TG(16:0/16:0/18:1)+NH4 | C53 H104 O6 N1 | 850.7858 | 21.3104 | 3.8179 | 4.8365 | 0.0396 |
| 439POS | TG(16:0/16:0/18:1)+NH4 | C53 H104 O6 N1 | 850.7858 | 22.4334 | 1.6947 | 11.5966 | 0.0356 |
| 440POS | TG(16:0/16:0/18:1)+NH4 | C53 H104 O6 N1 | 850.7858 | 22.7570 | 1.1415 | 8.3290 | 0.0297 |
| 442POS | TG(16:0/16:0/18:1)+NH4 | C53 H104 O6 N1 | 850.7858 | 21.6763 | 3.2852 | 6.8956 | 0.0288 |
| 444POS | TG(16:0/16:0/18:1)+NH4 | C53 H104 O6 N1 | 850.7858 | 23.7822 | 1.3571 | 13.1659 | 0.0460 |
| 445POS | TG(16:0/16:0/18:1)+NH4 | C53 H104 O6 N1 | 850.7858 | 25.8338 | 1.0613 | 5.7028 | 0.0328 |
| 469POS | TG(15:0/18:1/18:2)+NH4 | C54 H102 O6 N1 | 860.7701 | 19.8044 | 1.1627 | 3.8780 | 0.0406 |
| 479POS | TG(16:0/17:0/18:1)+NH4 | C54 H106 O6 N1 | 864.8014 | 21.6436 | 1.5521 | 4.3058 | 0.0411 |
| 481POS | TG(14:0/18:2/20:5)+NH4 | C55 H96 O6 N1 | 866.7232 | 16.8257 | 1.2285 | 9.7621 | 0.0196 |
| 486POS | TG(16:1/18:2/18:3)+NH4 | C55 H98 O6 N1 | 868.7388 | 17.2803 | 1.7230 | 3.2785 | 0.0378 |
| 487POS | TG(16:0/14:0/22:6)+NH4 | C55 H98 O6 N1 | 868.7388 | 18.2925 | 1.0232 | 8.7807 | 0.0050 |
| 490POS | TG(16:1/18:2/18:2)+NH4 | C55 H100 O6 N1 | 870.7545 | 18.8235 | 2.1759 | 4.8890 | 0.0205 |
| 492POS | TG(16:1/18:1/18:3)+NH4 | C55 H100 O6 N1 | 870.7545 | 18.1930 | 1.4737 | 4.0032 | 0.0424 |
| 495POS | TG(16:0/16:0/20:4)+NH4 | C55 H102 O6 N1 | 872.7701 | 20.0351 | 1.6847 | 4.2334 | 0.0189 |
| 501POS | TG(16:0/18:1/18:2)+NH4 | C55 H104 O6 N1 | 874.7858 | 21.6417 | 1.4180 | 7.0505 | 0.0421 |
| 528POS | TG(18:0/16:0/18:1)+NH4 | C55 H108 O6 N1 | 878.8171 | 22.7390 | 1.3026 | 5.7896 | 0.0148 |
| 536POS | TG(18:0/16:0/18:1)+NH4 | C55 H108 O6 N1 | 878.8171 | 22.2075 | 3.2297 | 4.7945 | 0.0442 |
| 561POS | TG(17:0/18:1/18:2)+NH4 | C56 H106 O6 N1 | 888.8014 | 20.8280 | 3.6276 | 2.2058 | 0.0325 |
| 572POS | TG(18:3/18:2/18:3)+NH4 | C57 H98 O6 N1 | 892.7388 | 17.3018 | 1.0584 | 6.4888 | 0.0087 |
| 574POS | TG(16:0/18:1/19:0)+NH4 | C56 H110 O6 N1 | 892.8327 | 22.4581 | 1.2444 | 4.0814 | 0.0418 |
| 583POS | TG(16:0/16:0/22:6)+NH4 | C57 H102 O6 N1 | 896.7701 | 19.5346 | 2.5469 | 7.1909 | 0.0043 |
| 587POS | TG(18:1/18:2/18:2)+NH4 | C57 H104 O6 N1 | 898.7858 | 19.3961 | 4.9433 | 2.7818 | 0.0494 |
| 598POS | TG(18:1/18:1/18:2)+NH4 | C57 H106 O6 N1 | 900.8014 | 21.6471 | 1.2570 | 5.7269 | 0.0400 |
| 618POS | TG(18:0/18:1/18:3)+NH4 | C57 H106 O6 N1 | 900.8014 | 20.5690 | 4.5542 | 2.6984 | 0.0275 |
| 684POS | TG(16:0/18:3/22:6)+NH4 | C59 H100 O6 N1 | 918.7545 | 17.6460 | 1.1743 | 5.6035 | 0.0072 |
| 685POS | TG(20:5/18:2/18:2)+NH4 | C59 H100 O6 N1 | 918.7545 | 17.0955 | 1.3005 | 2.7257 | 0.0312 |
| 686POS | TG(16:0/18:3/22:6)+NH4 | C59 H100 O6 N1 | 918.7545 | 17.3796 | 1.3397 | 5.5657 | 0.0049 |
| 689POS | TG(16:0/18:2/22:6)+NH4 | C59 H102 O6 N1 | 920.7701 | 18.5232 | 2.8692 | 6.2552 | 0.0076 |
| 692POS | TG(20:3/18:2/18:2)+NH4 | C59 H104 O6 N1 | 922.7858 | 18.6703 | 1.1780 | 3.8310 | 0.0168 |
| 694POS | TG(16:0/18:1/22:6)+NH4 | C59 H104 O6 N1 | 922.7858 | 19.5538 | 2.5840 | 6.4447 | 0.0050 |
| 697POS | TG(18:0/16:0/22:6)+NH4 | C59 H106 O6 N1 | 924.8014 | 20.7114 | 1.2744 | 5.3997 | 0.0057 |
| 698POS | TG(20:2/18:2/18:2)+NH4 | C59 H106 O6 N1 | 924.8014 | 19.6109 | 1.7419 | 2.5791 | 0.0005 |
| 699POS | DG(16:0/18:2)+NH4 | C37 H72 O5 N1 | 610.5405 | 12.1891 | 2.6714 | 1.5867 | 0.0139 |
| 703POS | TG(18:1/18:2/20:2)+NH4 | C59 H108 O6 N1 | 926.8171 | 20.5666 | 1.4089 | 2.9004 | 0.0339 |
| 744POS | TG(18:2/18:2/22:6)+NH4 | C61 H102 O6 N1 | 944.7701 | 17.5198 | 1.4967 | 4.8090 | 0.0065 |
| 749POS | TG(18:1/18:2/22:6)+NH4 | C61 H104 O6 N1 | 946.7858 | 18.5390 | 2.0805 | 5.9668 | 0.0078 |
| 767POS | TG(18:1/18:2/22:0)+NH4 | C61 H116 O6 N1 | 958.87 | 22.9134 | 1.2650 | 2.5673 | 0.0068 |
| 776POS | TG(16:0/22:6/22:6)+NH4 | C63 H102 O6 N1 | 968.7701 | 17.6593 | 1.2217 | 12.7397 | 0.0030 |
| 779POS | TG(22:5/18:2/20:4)+NH4 | C63 H104 O6 N1 | 970.7858 | 17.6351 | 1.0843 | 10.0591 | 0.0008 |
